# A Rare Intruder of the Liver: A Case of Undifferentiated Pleomorphic Sarcoma

**DOI:** 10.7759/cureus.80333

**Published:** 2025-03-10

**Authors:** Sidart Pradeep, Devine Thomas, Daniel Cain, Apurva Modi, Rohan Gupta, Shovendra Gautam

**Affiliations:** 1 Internal Medicine, Baylor Scott & White All Saints Medical Center, Fort Worth, USA; 2 Transplant Hepatology, Baylor Annette C. and Harold C. Simmons Transplant Institute, Baylor Scott & White All Saints Medical Center, Fort Worth, USA; 3 Hematology and Medical Oncology, The Center for Cancer and Blood Disorders, Fort Worth, USA

**Keywords:** liver lesion, obstructive jaundice, pleomorphic sarcoma, primary hepatic sarcoma, undifferentiated pleomorphic sarcoma of the liver, undifferentiated pleomorphic sarcoma (ups)

## Abstract

Undifferentiated pleomorphic sarcoma (UDS) is a rare and aggressive form of cancer that poses significant diagnostic and therapeutic challenges. The most common sites of this malignancy include the extremities. It is less common in visceral organs and the liver has rarely been reported as a primary site of disease. This subset of cancer is characterized by anaplastic and highly heterogeneous cells that lack specific markers of differentiation, making it difficult to identify and treat effectively. Due to vague symptoms that mirror other pathologies of the affected organ(s), UDS can present like various sarcomas or metastatic processes. A thorough microscopic analysis with the use of ancillary testing including immunohistochemistry can aid in the identification of UDS, as clinical symptoms can be non-specific. It is important to characterize primary malignant lesions since treatment options can range from medical therapy to surgical intervention. We present a unique case of UDS of the liver in a patient with obstructive jaundice.

## Introduction

Soft tissue sarcomas (STS) encompass a wide range of malignancies including undifferentiated pleomorphic sarcoma (UDS), previously known as malignant fibrous histiocytoma. Updated pathology analysis practices such as immunohistochemistry and cytogenetics reveal that many of these previously identified UDS were other types of sarcomas [1,2]. UDS is currently diagnosed once other STS have been excluded. 

Exploration of the pathogenesis of UDS has revealed that it can originate from a variety of cellular backgrounds and mutational aberrations [3]. Results from translational studies have implicated that dysregulation of secondary messengers in pathways such as Hippo, PIK3/PTEN/AKT/mTOR, and WNT/B-catenin may be involved [4-6]. The net effects of these molecular changes include unchecked cellular proliferation and impairment of transcriptional regulation. 

Although the mechanisms are poorly characterized, epidemiologic risk factors for UDS include male sex, White race, and age beyond the sixth decade of life [7]. Exposure to radiation therapy has been identified as a clinical risk factor for the development of UDS. In a large series of UDS cases, over 5% of cases were associated with a history of radiation with a median time of just over nine years between exposure to radiation and tumor development [8]. 

The clinical presentation of UDS can be ambiguous and vary depending on the site of involvement. The integumentary system is the most common site and often presents as asymptomatic cutaneous or subcutaneous nodules only rarely with abnormalities of the overlying skin [9]. Primary hepatic UDS is very uncommon and fewer than 200 cases have been reported globally [10]. It is thought that primary hepatic UDS accounts for just 0.1-2% of hepatic malignancies [11]. While there is a paucity of epidemiologic data regarding primary hepatic UDS, a series of 100 UDS cases at a large hospital reported none originating from the liver [12]. Internal organ involvement typically results in mass effect of adjacent structures. Therapeutic intervention can include surgery, radiation therapy, antineoplastic chemotherapy, and immunotherapy depending on the site and extent of involvement. 

## Case presentation

A 72-year-old male patient with a history of atrial fibrillation on anticoagulation, congestive heart failure, coronary artery disease, and hepatitis C status post sustained virologic response presented to an outside facility emergency room with new jaundice of the skin and malaise ongoing for two weeks prior to presentation. Initial documented evaluation at the outside facility revealed normal vital signs, jaundice of the skin, conjunctival icterus, and generalized abdominal distention. Mental status was at baseline and no asterixis was documented as present. Associated symptoms at the initial exam there included 20-25-pound unintentional weight loss over the previous two months, night sweats, and clay-colored stools. He had denied any history of chronic liver disease or alcohol use. He had no previous exposure to radiation therapy.

Initial outside facility labs (Table 1) were notable for coagulopathy and liver dysfunction. A comprehensive metabolic panel demonstrated elevated total bilirubin, alkaline phosphatase (ALP), aspartate aminotransferase (AST), alanine aminotransferase (ALT), low albumin, and normal renal function. Platelets were in the normal range. The international normalized ratio (INR) was elevated at 1.4 while prothrombin (PT) and activated partial thromboplastin (APTT) times were elevated at 17.2 seconds and 40.7 seconds, respectively. The model for end-stage liver disease-Na (MELD-Na) score was 26. 

**Table 1:** Laboratory test results at presentation at the outside facility and at arrival at our facility

Lab Study	Results at the Outside facility	Results at Our Facility	Reference Range
Total Bilirubin	13.7 mg/dL	22.0 mg/dL	
Alkaline Phosphatase (ALP)	410 U/L	379 U/L	45-117 U/L
AST (SGOT)	118 U/L	134 U/L	15-37 U/L
ALT (SGPT)	37 U/L	35 U/L	16-61 U/L
Total Protein	8.2 U/L	8.1 g/dL	6.4-8.2 g/dL
Albumin	2.5 g/dL	1.6 g/dL	3.4-5.0 g/dL
White Blood Cell (WBC)	19.5 K/uL	18.6 K/uL	4.5-11.0 K/uL
Hemoglobin	11.9 g/dL	11.5 g/dL	13.5-18.0 g/dL
Hematocrit	35.60%	34.70%	40.0-52.0%
Platelets	225 K/uL	258 K/uL	140-440 K/uL
Prothrombin Time (PT)	17.2 sec	25.8 sec	9.4-12.5 sec
International Normalized Ratio (INR)	1.4	2.2	
Partial Thromboplastin Time (aPTT)	40.7 sec	46.8 sec	25.1-36.5 sec
Hepatitis A IgM	Non-Reactive		
Hepatitis B Core IgM	Non-Reactive		
Hepatitis Bs Ag	Non-Reactive		
Hepatitis C Antibody	Non-Reactive		
Anti-Mitochondrial Antibody (AMA)	Negative		
Anti-Smooth Muscle Antibody (ASMA)	1:320		
Alpha Fetoprotein (AFP)	1.7 ng/mL		<6.1 ng/mL
Carcinoembryonic Antigen (CEA)	1.6 ng/mL		0.0-2.4 ng/mL
CA 19-9	53 U/mL		<34 U/mL

**Figure 1:**
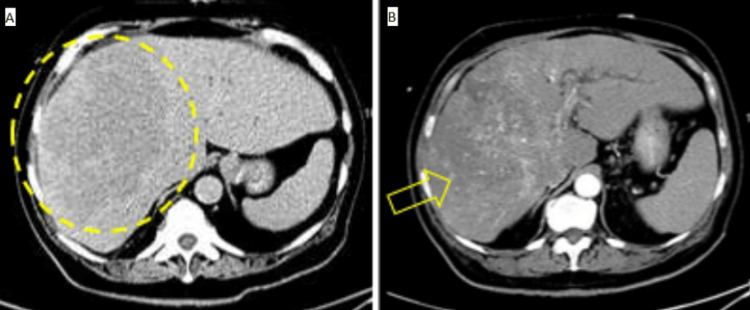
CT of the abdomen and pelvis with (A) and without (B) intravenous contrast demonstrating the hypervascular mass in the right hepatic lobe.

Computed tomography (CT) of the abdomen and pelvis with contrast demonstrated a large hypervascular mass centered in the right hepatic lobe with invasion of the left hepatic lobe (Figure 1). There was an associated tumor thrombus in the right and left portal veins along with moderate intrahepatic biliary obstruction and dilatation by the mass. A CT of the chest with contrast revealed an enlarged posterior mediastinal/retrocrural lymph node adjacent to the esophagus. These findings were concerning for malignancy.

**Figure 2:**
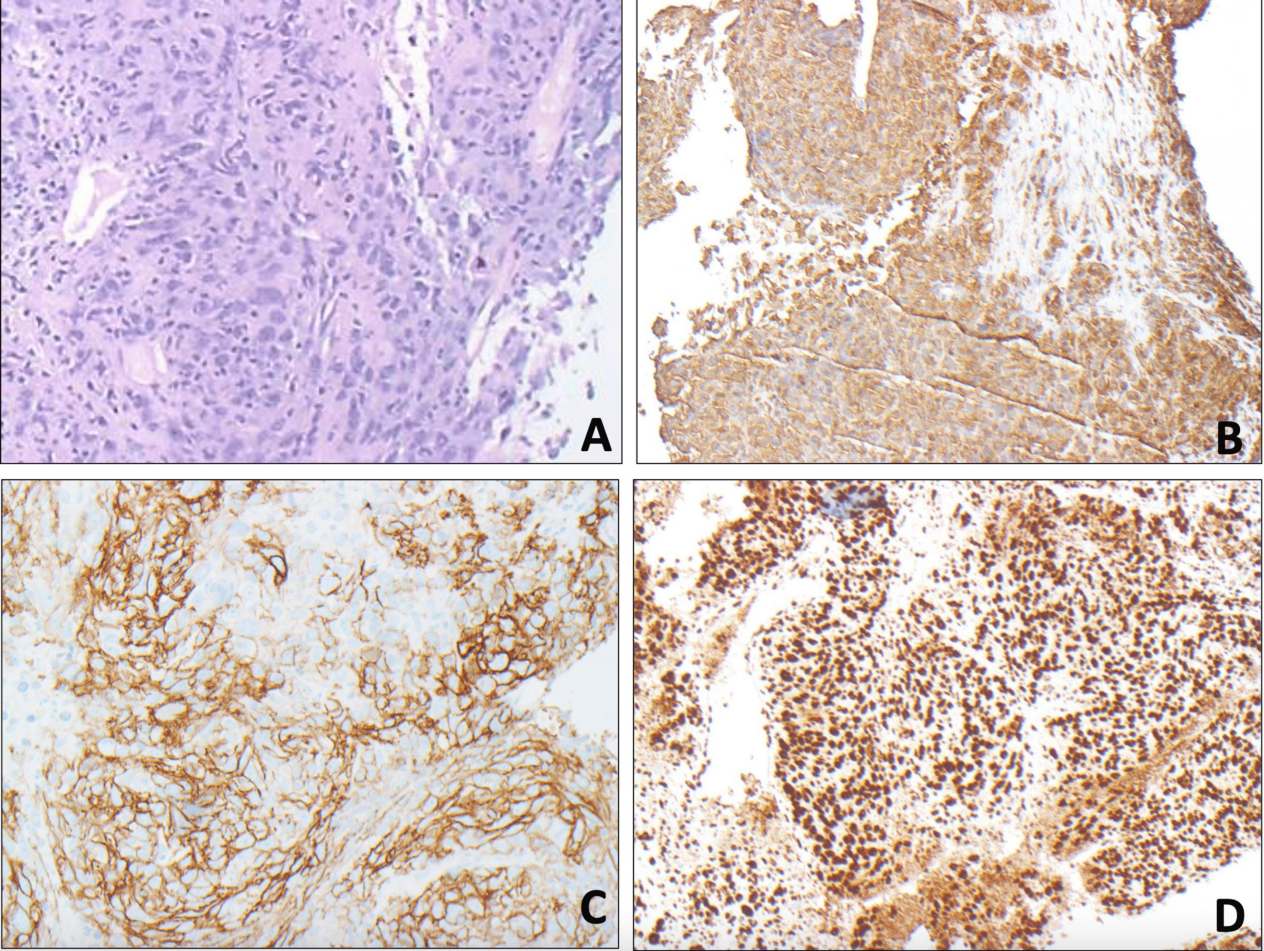
Specialty pathology analysis. Immunohistochemical stains were performed on biopsy samples showing vimentin (B), CD23 (C), and PMS2 (D) positivity, which indicates a high-grade nonepithelial neoplasm.

A liver biopsy of the hepatic mass was performed at the outside facility. Biopsy samples underwent histologic and immunohistochemical (IHC) analysis. Samples were positive for D2-40, vimentin, PMS2, MSH6, were weakly positive for CD21, and diffusely positive for CD23 (Figure 2). They were negative for other relevant IHC stains including AE1/3, CK7, CK18, CK20, CK5/6, hepatocyte-specific antigen (HSA), glypican 3, arginase 1, CDX2, TTF1, S100, epithelial cell adhesion molecule (EpCAM), Ber-EP4, PSA, PSAP, CD34, CD35, CD68, CD117, smooth muscle actin (SMA), GATA3, WT1, OSCAR, epithelial membrane antigen (EMA), desmin, fascin. Histologic evaluation revealed cells with markedly enlarged pleomorphic nuclei with vesicular chromatin, prominent nucleoli, and variable amounts of cytoplasm. These findings were consistent with high-grade sarcoma/mesothelioma and suggestive of UDS. For diagnostic confirmation, the specimen was sent to the Mayo Clinic for specialty pathology analysis.

Due to worsened hepatic decompensation and overall clinical worsening, the patient was transferred to our facility for a higher level of care after 10 days. On arrival at our center, physical exam was notable for conjunctival icterus, jaundice of the skin and oral mucosa, and generalized abdominal distention with no shifting dullness. Laboratory test results (Table 1) showed elevated ALP, total bilirubin, AST, low albumin, and mild hyponatremia. Coagulation studies were notable for an INR of 2.2, PT of 25.8 seconds, and aPTT of 46.8 seconds. MELD-Na score at that time was 31. 

The serologic screening was negative for viral hepatitis. Alpha-fetoprotein and carcinoembryonic antigen were negative, while cancer antigen (CA)19-9 levels were mildly elevated. Specialty pathology analysis from the Mayo Clinic returned and revealed the identity of the tumor as UDS. Due to the unresectable nature of the disease in this patient and poor clinical status, it was determined he was not a suitable candidate for surgery, chemotherapy, or immunotherapy. Since there was visceral organ involvement, radiation therapy was not a viable option. No further interventions were pursued as the patient opted for inpatient hospice. 

## Discussion

UDS has a non-specific appearance on imaging requiring the use of IHC for diagnosis. Due to a lack of reliable markers of identification, UDS is typically a diagnosis of exclusion and there is much controversy surrounding the diagnosis [13]. CA 19-9 was only mildly elevated in the current and this is consistent with the tumor thrombus seen in the bilateral portal veins as well as the tumor burden present on discovery. Current literature and experts vacillate between two schools of thought. One suggests that UDS is a common morphologic pattern of many different neoplasms and the other proposes that UDS originates from mesenchymal stem cells [14,15]. To make the diagnosis, it is suggested that other malignancies must be excluded with comprehensive immunohistochemical analysis [16,17]. Testing for mismatch repair protein deficiencies such as PMS2 and MSH6 can indicate microsatellite instability and the presence of cancer predisposition syndromes in the context of known sarcoma [17,18]. 

Currently, there are sparse treatment guidelines for UDS. En-bloc resection of UDS of the head, neck, trunk, and extremities with negative margins on microscopy is considered the standard of care for appropriate lesions. Resection sites with positive microscopic margins, resection margins within 1 cm of the tumor, and involvement of bone, vasculature, or nerve structures are appropriate for radiation therapy [19]. Other options with mixed evidence include Mohs surgery with a last alternative being extremity amputation. 

A branch point in therapy is the presence of visceral organ involvement, which is typically unresectable and disqualifies the disease from surgical therapy. Standard chemotherapy increases disease-free survival, with a randomized controlled trial indicating a preferred regimen of anthracycline with ifosfamide [20]. The efficacy of immune checkpoint inhibitor therapies such as anti-PD1, anti-CTLA4, anti-NTSR1, anti-PI3K, anti-mTOR, and anti-IGF1R therapies is currently being studied. Some evidence suggests that a combination of chemotherapy and immunotherapy may be a successful strategy in some patients [21-24]. 

A typical histologic appearance of UDS includes atypical pleomorphic cells with mitotic figures, although these are relatively nonspecific findings [19]. IHC stains for LN2, vimentin, p53, and D2-40 may be positive. The definitive diagnosis is from the exclusion of other malignancies with a complete IHC panel [18], as was done in this patient. 

## Conclusions

In patients presenting with an obstructive mass in the liver, diligent pathologic analysis is of paramount importance. While UDS in the liver is rare, it should be considered on the differential if biopsy analysis suggests a sarcoma/mesothelioma-like process. Multi-disciplinary investigation should be pursued with liver biopsy being the most likely modality to elucidate a diagnosis. Due to its rarity and the lack of established treatment protocols, there is a need for greater characterization of UDS to shed light on the clinical features, diagnostic tools, and treatment options available for this aggressive malignancy. Our case emphasizes the importance of specialized pathology analysis and the rarity of primary hepatic UDS. 
